# Hypoxia-induced NFATc3 deSUMOylation enhances pancreatic carcinoma progression

**DOI:** 10.1038/s41419-022-04779-9

**Published:** 2022-04-28

**Authors:** Yingying Tong, Zheng Zhang, Yurong Cheng, Jing Yang, Cong Fan, Xuyang Zhang, Jiandong Yang, Li Wang, Dong Guo, Dong Yan

**Affiliations:** 1 Cancer Center, Beijing Luhe Hospital, Capital Medical University, Beijing, 101149 China; 2 Department of Gastroenterology, Beijing Friendship Hospital, Capital Medical University, National Clinical Research Center for Digestive Disease, Beijing Digestive Disease Center, Beijing Key Laboratory for Precancerous Lesion of Digestive Disease, Beijing, 100050 China; 3 Zhejiang Provincial Key Laboratory of Pancreatic Disease, The First Affiliated Hospital, and Institute of Translational Medicine, Zhejiang University School of Medicine, Zhejiang University, Hangzhou, Zhejiang 310029 China; 4 Zhejiang University Cancer Center, Hangzhou, Zhejiang 310029 China

**Keywords:** Gastrointestinal cancer, Cell signalling

## Abstract

The transcriptional regulator nuclear factor of activated T-cells, cytoplasmic 3 (NFATc3) is constitutively activated in several cancer types and plays important roles in cancer development and progression. Heavily phosphorylated NFATc3 resides in the cytoplasm of resting cells, and dephosphorylated NFATc3 translocates to the nucleus to activate expression of target genes in cells exposed to stimuli, for instance, hypoxia. Apart from phosphorylation, various post-translational modifications have been reported to regulate NFAT transcriptional activity. However, the mechanisms remain elusive. Here, we have demonstrated that NFATc3 is activated in human pancreatic ductal adenocarcinoma (PDAC) cells and that excessive activation of NFATc3 is correlated to advanced stages of PDAC and short survival time of PDAC patients. NFATc3 is deSUMOylated at K384 by SENP3 under hypoxia, which impairs the interaction between NFATc3 and phosphokinase GSK-3β, subsequently decreases NFATc3 phosphorylation and increases its nuclear occupancy. Knockdown of SENP3 greatly decreased hypoxia-induced NFATc3 nuclear occupancy. Our results highlight that SENP3-mediated deSUMOylation acts as an essential modulator of NFATc3, which is instrumental in PDAC tumor progression under hypoxia.

## Introduction

The nuclear factor of activated T-cells (NFAT) family of transcription factors has been identified as an inducible nuclear factor that plays important roles in regulating the transcription of cytokines [1]. The NFAT family contains five members, including genuine Ca^2+^-dependent isoforms named NFAT1 (NFATc2), NFAT2 (NFATc1), NFAT3 (NFATc4), and NFAT4 (NFATc3), and a tonicity-responsive enhancer-binding protein (TonEBP, also known as NFAT5) [2]. The classic NFAT isoforms (NFAT1–NFAT4) exist in a hyperphosphorylated state in the cytoplasm under resting conditions. They are usually activated by increased intracellular calcium levels, via dephosphorylation by the phosphatase calcineurin, which triggers the transport of NFAT proteins from the cytoplasm to the nucleus. Once in the nucleus, NFAT proteins collaborate with other factors to activate transcription of downstream genes, which are essential for many biological functions [3]. To counteract NFAT proteins dephosphorylation and nuclear localization, several maintenance kinases including dual-specificity tyrosine-phosphorylation regulated kinase 1/2 (DYRK1 and DYRK2), casein kinase 1 (CK1) and glycogen synthase kinase 3 (GSK-3) act to phosphorylate the NFAT proteins at the N-terminus to reduce their nuclear occupancy by either increasing their nuclear export or decreasing their nuclear import [4]. The balance between cytoplasmic/nuclear trafficking of NFAT proteins is critical for NFATs activity regulation. Additional mechanisms that regulate the nuclear translocation of NFATs still remain to be investigated, which would facilitate the development of new treatment strategies for various diseases and help minimize side effects.

The NFAT isoforms are constitutively activated in several tumors. It is believed that they are associated with cancer development and progression. The NFAT proteins have been conclusively proved to be pivotal in cell survival, differentiation, angiogenesis, invasion, and migration [5, 6]. In particular, the latest research shown that constitutively activation of NFATc3 isoform was associated with intestinal tumor progression [7, 8]. However, the role of NFATc3 in pancreatic ductal adenocarcinoma (PDAC) remains unclear. PDAC has a universally poor prognosis and is predicted to be the second leading cause of cancer death by 2030 [9]. The main feature of PDAC is abundant fibrosis and extracellular matrix deposition, which results in limited blood flow to the tumor and consequent hypoxia [10]. In turn, the oncogenic pathway to support tumor cell growth in the hypoxia microenvironment is found activated in PDAC cells [11]. Recent studies have further indicated that hypoxia can induce the activation of NFATc3 in colon cancer cells via upregulation of Orai1, which leads to increased intracellular Ca^2+^ and NFATc3 nuclear anchorage, resulting in the transcriptional activation of downstream genes [12]. However, key components and molecular mechanisms contributing to the activation of NFATc3 in PDAC still remain unclear.

Apart from phosphorylation, various post-translational modifications of NFAT proteins have been reported, and SUMOylation is one example [13]. Similar to ubiquitination, SUMOylation is an important reversible post-translational protein modification, catalyzed by activating (E1), conjugating (E2), and ligating (E3) enzymes [14]. DeSUMOylation is catalyzed by SUMO-specific protease in humans (SENPs, SENP1–SENP3, and SENP5–SENP7.) [15–17]. Most of these SENPs concentrate in the nucleus, with many of them localizing to recognizable subnuclear compartments. Among SENPs, SENP3 localizes to the nucleolus and has substrate-specificity with a preference for SUMO2 or SUMO3 [18]. In tumor cells, the stability of SENP3 protein increases and accumulates in the nucleoplasm by altering redox state under hypoxia, oxidative stress and other stimuli [19–21]. Research have revealed that increased SENP3 regulates the functions of its substrates in cancer cells, leading to enhanced tumorigenesis, cell proliferation, epithelial–mesenchymal transition, and angiogenesis [22]. Here, we demonstrate that NFATc3 is mainly located in the nucleus in PDAC specimens and associated with advanced stages of PDAC and low survival rates in PDAC patients. Exposure of PDAC cells to hypoxia induced rapid translocation of NFATc3 into the nucleus, accompanied by an increase of SENP3. In the meantime, the positive intensity of SENP3 proteins and NFATc3 nuclear occupancy in the same areas within one PDAC specimen also displayed a linear correlation. SENP3 could de-conjugating SUMO3 modification at lysine 384 of NFATc3. This deSUMOylation reduces binding of NFATc3 with its phosphokinase GSK-3 in the nucleus, leading to decreased nuclear export. Thus, SENP3-mediated NFATc3 deSUMOylation could increase NFATc3 nuclear occupancy and activate NFATc3 signaling for promoting tumor cell proliferation and invasion under hypoxia.

## Results

### Nuclear localized NFATc3 is positively correlated with Hif1α and poorer prognosis in PDAC specimens

**Fig. 1:**
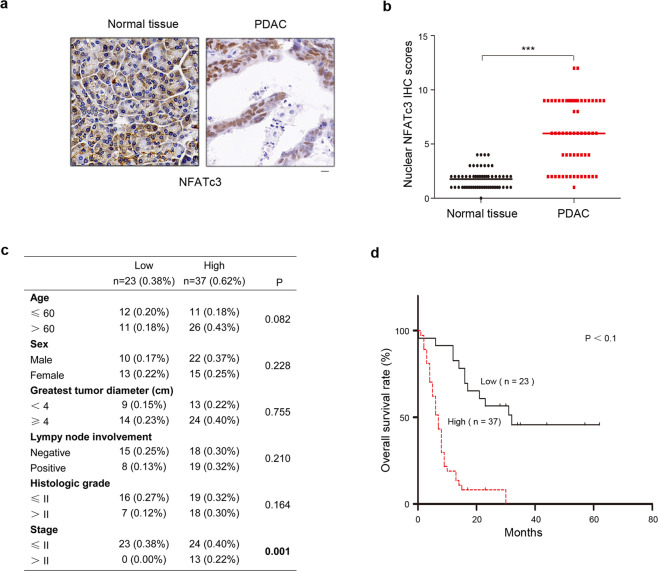
NFATc3 is mainly located in the nucleus in PDAC specimens and indicates poorer prognosis. **a** Expression and location of NFATc3 in 60 samples of human PDAC tissues and matched adjacent normal tissues by IHC staining with an anti-NFATc3 antibody. Representative images are shown. Scale bars, 10 μm. **b** Comparative analysis of nuclear NFATc3 expression between PDAC tissues and matched adjacent normal tissues (***P < 0.001). **c** Correlations between nuclear NFATc3 expression levels and PDAC clinicopathological parameters. **d** Kaplan–Meier plots and *p*-values of the log-rank test for comparing survivals of PDAC patients with high (staining score, 5–12) and low (staining score, 0–4) expression of nuclear NFATc3.

To determine the expression pattern and localization of NFATc3 in PDAC, we performed immunohistochemical (IHC) assays in 60 pairs of primary PDAC tumor specimens and their matched normal tissues with the anti-NFATc3 antibody. Then, we analyzed the correlation between NFATc3 nuclear occupancy and clinical feature of PDAC patients. IHC staining results suggested that NFATc3 was mainly expressed in the nucleus in primary PDAC tumor specimens, and in the cytoplasm in the matched normal tissues, respectively (Fig. 1a, b). The levels of NFATc3 nuclear occupancy were positively correlated to advanced TNM stages of PDAC (*P* < 0.001) (Fig. 1c). In addition, we assessed the survival durations of the 60 PDAC patients corresponding to these obtained samples. Kaplan–Meier analysis showed that high nuclear expression of NFATc3 was significantly associated with worse survival (Fig. 1d). These results demonstrated an increased nuclear localization of NFATc3 in PDAC tumor specimens and suggested that it may help predict the aggressiveness of human PDAC.

PDAC is characterized by hypovascularization and extensive fibrosis, resulting in significant intratumoral hypoxia that activates other signaling pathways and factors and contributes to its aggressiveness and high mortality [10]. As expected, our IHC staining analysis indicated hypoxia-inducible factor 1 alpha (HIF1α) displayed a tendency of upregulated-expression in tumor tissues compared with adjacent normal tissues (Fig. S1a, b). Kaplan–Meier analysis showed that high expression of HIF1α was significantly associated with worse survival (Fig. S1c). Meanwhile, HIF1α expression was positively correlated with the levels of NFATc3 nuclear occupancy in the PDAC tissues (Fig. S1d). These data suggest that the positive correlation of nuclear NFATc3 and hypoxia in PDAC specimens.

### Hypoxia activates NFATc3 signaling in PDAC cells

**Fig. 2:**
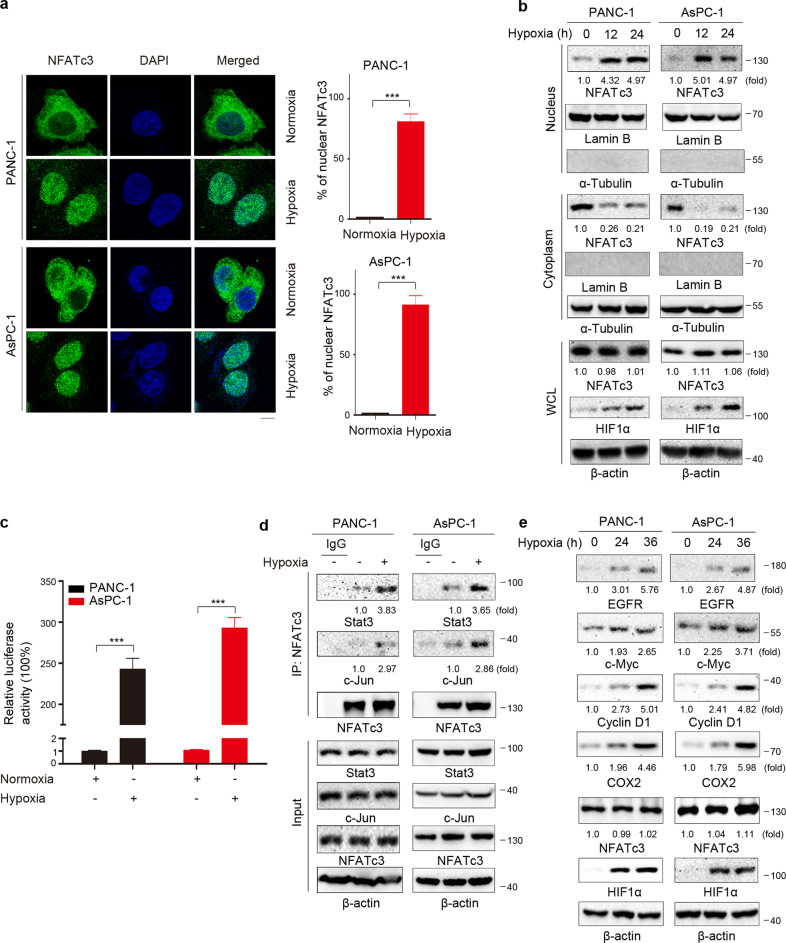
Hypoxia activates NFATc3 signaling in PDAC cells. **a** PANC-1 and AsPC-1 cells were stimulated with or without hypoxia for 12 h. Immunofluorescent analyses were performed with an anti-NFATc3 antibody, and the percentage of nuclear NFATc3 was quantitated (right) using ImageJ. Scale bars, 10 μm. **b** PANC-1 and AsPC-1 cells were stimulated with or without hypoxia for the indicated time. Cytosolic and nuclear fractions were prepared, and immunoblotting analyses with the indicated antibodies were performed. **c** PANC-1 and AsPC-1 cells were transfected with luciferase reporter gene plasmid (NFATc3-Luc) and were stimulated with or without hypoxia for 12 h. The data are presented as the mean ± SD of triplicate samples (***P < 0.001). **d** PANC-1 and AsPC-1 cells were stimulated with or without hypoxia for the 12 h. Immunoprecipitation with an anti-NFATc3 antibody was performed. **e** PANC-1 and AsPC-1 cells were stimulated with or without hypoxia for the indicated time. Immunoblot analyses were performed with the indicated antibodies.

It was recently reported that hypoxia could trigger the activation of NFATc3 in colon cancer cells [12]. To determine whether hypoxia enhances nuclear localization of NFATc3 in PDAC cells, we examined the cellular distribution of NFATc3 in PDAC cells upon hypoxia stimulation. Immunofluorescence (IF) analyses of PANC-1 and AsPC-1 human PDAC cells demonstrated a hypoxia**-**induced nuclear accumulation of NFATc3 (Fig. 2a). Cell fractionation analysis showed that hypoxia led to the translocation of most cytosolic NFATc3 into the nucleus (Fig. 2b). In addition, we found that the total protein level of NFATc3 was not affected by hypoxia in either PANC-1 or AsPC-1 cells (Fig. 2b).

Nuclear localization of NFATc3 is required for its transcriptional activity. A luciferase activity assay demonstrated that hypoxia induced NFATc3 activation in PANC-1 and AsPC-1 cells (Fig. 2c). Previous studies indicated that once in the nucleus, NFATc3 can collaborate with other factors to coordinate target gene expression, which are essential for many biological functions [5]. Therefore, we measured the interaction between NFATc3 and stat3 or AP-1. Immunoprecipitation analyses demonstrated that hypoxia stimulation notably increased the interaction between NFATc3 and stat3 or AP-1 in both PANC-1 or AsPC-1 cells (Fig. 2d). In addition, hypoxia stimulation significantly increased the expression of NFATc3 target genes at mRNA and protein levels (Fig. S1e and Fig. 2e). These results indicated that hypoxia stimulation could induce the nuclear accumulation of NFATc3 without affecting NFATc3 protein level, and promote the transcriptional activity of NFATc3 in PDAC cells.

### Hypoxia induces NFATc3 K384 deSUMOylation

**Fig. 3:**
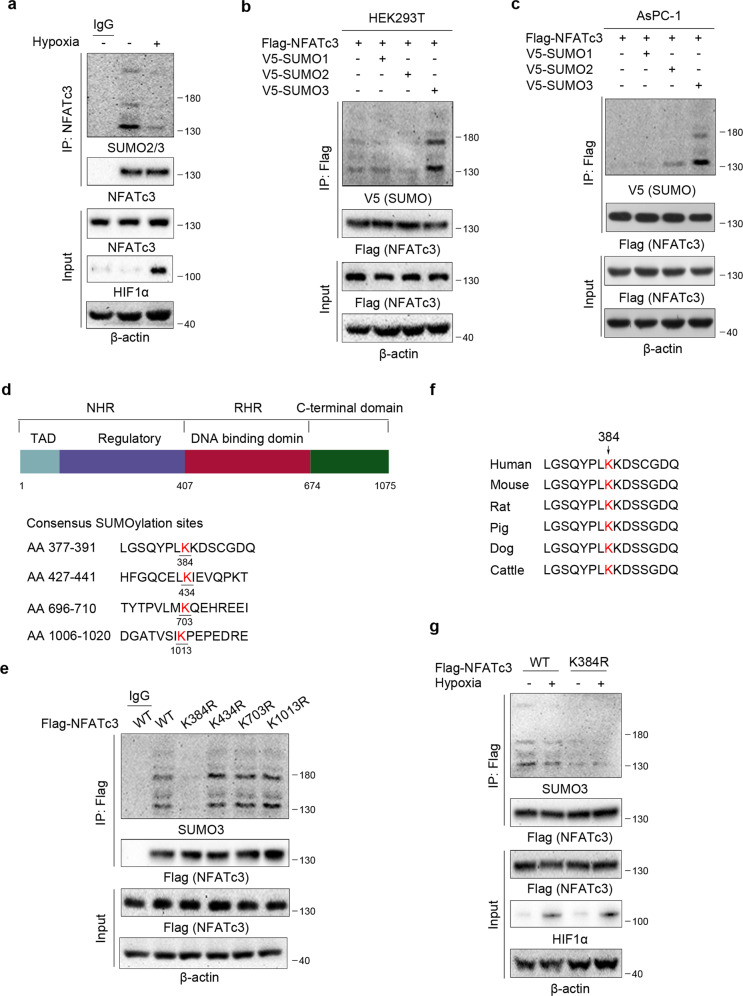
Hypoxia induces NFATc3 K384 deSUMOylation. **a**, **b**, **c**, **e**, **g** Immunoprecipitation and immunoblot analyses were performed with the indicated antibodies. **a** AsPC-1 cells were stimulated with or without hypoxia for 12 h. **b**, **c** HEK293T cells (**b**) and AsPC-1 cells (**c**) were transfected with Flag-NFATc3, V5-SUMO1,V5-SUMO2, or V5-SUMO3 plasmids as indicated. **d** Sketch of NFATc3 Structural Features (upper panel). NHR, NFAT homology region; RHR, Rel homology region; TAD, transactivation domain. The potential SUMOylation sites in NFATc3 were shown (lower panel). **e** Flag-WT NFATc3 or mutants (384, 434, 703, and 1013) were expressed in HEK293T cells. **f** Lys384 of NFATc3 is evolutionarily conserved in the indicated species. Alignment of the sequences around NFATc3 K384 is illustrated. **g** AsPC-1 cells expressing WT NFATc3 or NFATc3 K384R were cultured for 24 h under normoxia or hypoxia condition.

It was recently reported that hypoxia could trigger the activation of NFATc3 via upregulation of Orai1, which leads to increased intracellular Ca^2+^ and the following activation of calcineurin, resulting in NFATc3 dephosphorylation and nuclear import in colon cancer cells [12]. However, whether and how other post-translational modifications regulate NFATc3 nuclear translocation remains elusive. SUMO modification of transcription factors is often associated with the inhibition of target gene expression through multifaceted mechanisms under hypoxia. Therefore, we examined whether hypoxia could regulate NFATc3 nuclear translocation through altering SUMO modification of NFATc3. Using a pan-SUMO2/3 antibody, it is observed that hypoxia stimulation could decrease NFATc3 SUMOylation in AsPC-1 cells (Fig. 3a). In mammalian cells, three SUMO forms (SUMO1, SUMO2, and SUMO3) act as covalent modifiers of target proteins. As shown in Fig. 3b, NFATc3 is mostly modified by SUMO3 in HEK293T cells. The same result was observed in AsPC-1 human PDAC cells (Fig. 3c). According to the JASSA Analysis Programs, GPS-SUMO, and SUMOplot™ database, we have found that NFATc3 contains four high scoring sites (384, 434, 703, and 1013) as possible SUMO modification sites in its amino acid sequence (Fig. 3d). To determine which lysine residue(s) is the major SUMOylation site(s), we constructed wild-type (WT) NFATc3 and deSUMOylation-mimic NFATc3 mutant in which the predicted four putative Lys residues were mutated to Arg individually. Among those four mutants, only the mutation of evolutionarily conserved K384R (Fig. 3f) notably reduced SUMOylation of NFATc3 (Fig. 3e), suggesting that K384 could be the major SUMOylation site of NFATc3. Importantly, a substantial decrease of NFATc3 SUMOylation was detected, while no obvious change of NFATc3 K384R SUMOylation was observed in AsPC-1 cells under hypoxia (Fig. 3g). These data showed that K384 SUMOylation of NFATc3 occurred under physiological conditions and could be deSUMOylated under hypoxia.

### NFATc3 is deSUMOylated by SENP3 under hypoxia

**Fig. 4:**
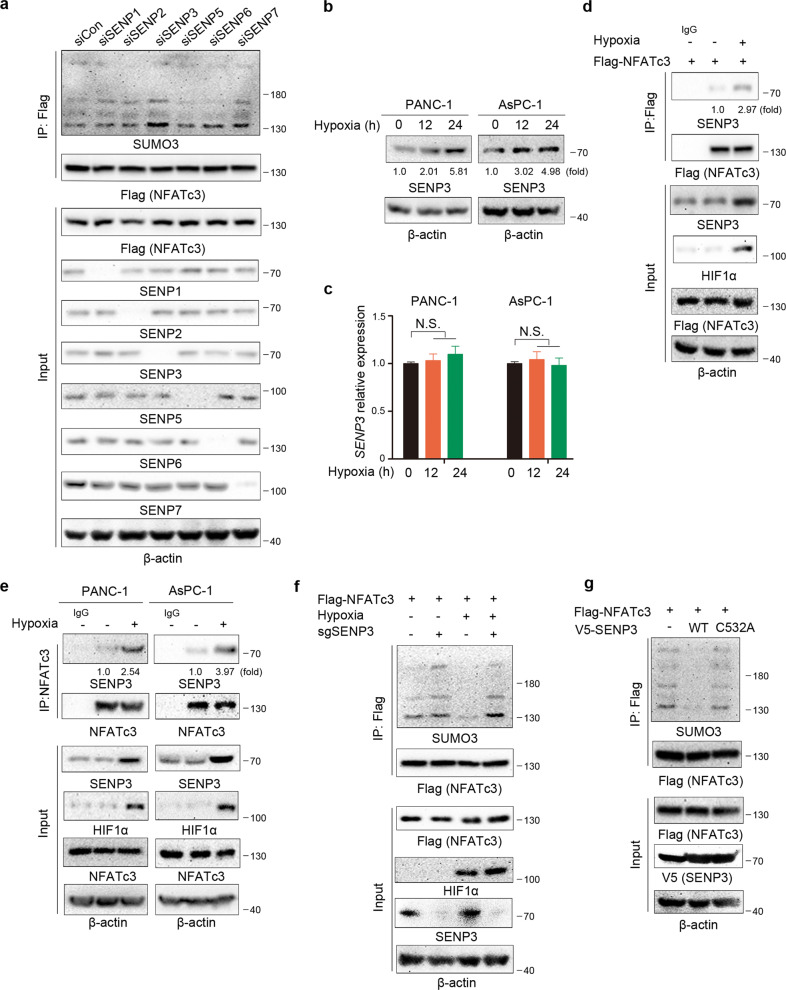
NFATc3 is deSUMOylated by SENP3 under hypoxia. **a, d, e, f,** **g** Immunoprecipitation and immunoblot analyses were performed with the indicated antibodies. **a** AsPC-1 cells that stably expressed Flag-NFATc3 were transfected with non-specific siRNA (siCon) or SENPs siRNA for 72 h. **b**, **c** PANC-1 and AsPC-1 cells were cultured under normoxia or hypoxia condition for the indicated time. Immunoblot analyses (**b**) and RT-PCR (**c**) were performed (N.S. = not significant for the indicated comparison). **d** AsPC-1 cells that stably expressed Flag-NFATc3 were cultured under normoxia or hypoxia condition for 24 h. **e** PANC-1 and AsPC-1 cells were cultured under normoxia or hypoxia condition for 24 h. **f** AsPC-1 cells with or without SENP3 depletion and reconstituted expression of Flag-NFATc3 were cultured for 24 h under normoxia or hypoxia. **g** AsPC-1 cells that stably expressed Flag-NFATc3 were transfected with V5-WT SENP3 or V5-SENP3 C532A plasmids as indicated.

It was reported that cysteine proteases of the sentrin-specific protease (SENP, SENP1-3, and SENP5-7) family could reverse SUMO conjugation in mammalian cells [23, 24]. To determine which SUMO protease is involved in NFATc3 K384 deSUMOylation, we used small interfering RNA (siRNA) directed against SENP1-3 and SENP5-7 and observed whether NFATc3 K384 SUMOylation was affected after knocking down any specific SENPs. We found that knockdown of SENP3, but none of the other SENPs, increased the SUMOylation of NFATc3 (Fig. 4a). These data suggested that NFATc3 might be a substrate of SENP3. Moreover, in line with previous findings, hypoxia led to a significant increase of SENP3 protein levels in both PANC-1 and AsPC-1 cells (Fig. 4b), without altering its mRNA expression (Fig. 4c). Correspondingly, we revealed that the levels of SENP3 were markedly higher in the PDAC samples than in their adjacent normal tissues (Fig. S2a, b) and HIF1α and SENP3 were correlated with each other in PDAC samples (Fig. S2c). Of note, high levels of SENP3 in the PDAC samples appeared to have the worse prognosis (Fig. S2d). Furthermore, we examined the interaction between NFATc3 and SENP3 in both PANC-1 and AsPC-1 cells, and results showed that they interacted extensively in these two cell lines under hypoxia (Fig. 4e). In AsPC-1 cells transfected with Flag-tagged NFATc3, we found that hypoxia greatly enhanced NFATc3-SENP3 interaction (Fig. 4d). In addition, hypoxia led to a remarkable decrease of NFATc3 SUMOylation, which could be reversed by further knockout of SENP3 by a sgRNA-targeting SENP3 (Fig. 4f). Consistently, overexpression of WT SENP3 was able to remove SUMO3 from NFATc3, whereas the SENP3 mutant (C532A, loss of enzyme activity) was not capable of doing so (Fig. 4g). These data indicated that SENP3 could induce NFATc3 deSUMOylation under hypoxia.

### NFATc3 K384 deSUMOylation by SENP3 increases nuclear translocation of NFATc3 by decreasing its interaction with GSK-3β

**Fig. 5:**
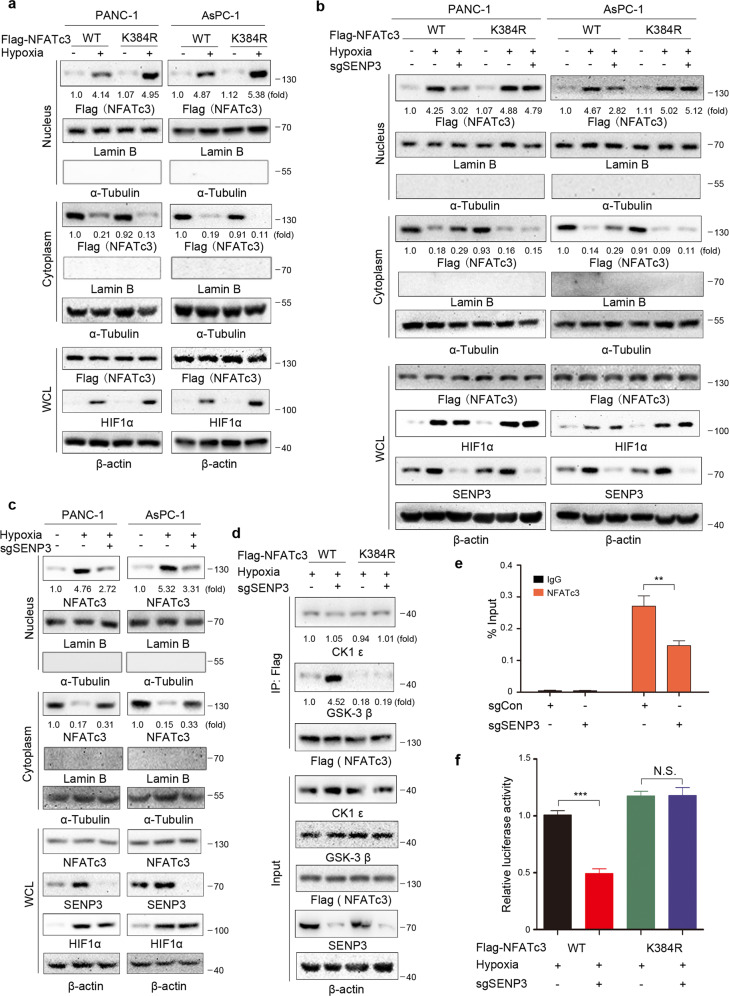
NFATc3 K384 deSUMOylation by SENP3 increases nuclear translocation of NFATc3 by decreasing its interaction with GSK-3β. **a**–**c** Cytosolic and nuclear fractions were prepared and immunoblotting analyses with the indicated antibodies were performed. a. PANC-1 and AsPC-1 cells that stably expressed Flag-WT NFATc3 or Flag-NATc3 K384R were cultured under normoxia or hypoxia condition for 24 h. **b** PANC-1 and AsPC-1 cells with or without the expression of SENP3 sgRNA expressed Flag-WT NFATc3 or Flag-NFATc3 K384R proteins. The indicated cells were cultured under normoxia or hypoxia condition for 24 h. **c** Parental and the indicated SENP3-knockout AsPC-1 and PANC-1 cells were cultured under normoxia or hypoxia condition for 24 h. **d** AsPC-1 cells with or without the expression of SENP3 sgRNA expressed Flag-WT NFATc3 or Flag-NFATc3 K384R proteins. Immunoprecipitation and immunoblot analyses were performed with the indicated antibodies. **e** ChIP assay with an anti-NFATc3 antibody and quantitative PCR with primers against the promoter regions of *MYC* in AsPC-1 cells with or without SENP3 depletion were performed. Two-sided *t*-test analyses were conducted. The data are presented as the means ± S.D. of three independent experiments (*n* = 3). **P < 0.01. **f** Flag-WT NFATc3 or Flag-NFATc3 K384R was expressed in AsPC-1 and PANC-1 cells with or without the expression of SENP3 sgRNA. The indicated cells were transfected with luciferase reporter plasmid (NFATc3-Luc) and stimulated with or without hypoxia for 12 h. The data are presented as the mean ± SD of triplicate samples (***P < 0.001; N.S. = not significant for the indicated comparison).

To further elucidate the relationship between NFATc3 K384 deSUMOylation and its nuclear translocation, we treated PANC-1 and AsPC-1 cells with hypoxic stimulation for 24 h. Cell fractionation analysis demonstrated that, compared with the WT NFATc3 control, the nuclear translocation of the deSUMOylation-mimic Flag-NFATc3 K384R mutant was slightly increased under hypoxia in PANC-1 and AsPC-1 cells, reflecting the inverse relationship of NFATc3 SUMOylation with nuclear translocation (Fig. 5a). Next, we found that depletion of SENP3, which de-conjugated SUMO at lysine 384 of NFATc3, decreased the hypoxia-induced nuclear translocation of WT NFATc3. However, SENP3 deficiency did not block hypoxia-induced nuclear translocation of NFATc3 K384R (Fig. 5b). These results were further supported by cell fractionation analysis, which showed that SENP3 deficiency decreased nuclear translocation of endogenous NFATc3 under hypoxia stimulation in PANC-1 and AsPC-1 cells (Fig. 5c). In the meantime, the positive intensity of SENP3 proteins and NFATc3 nuclear occupancy in the same areas within one PDAC specimen also displayed a linear correlation (Fig. S3a). To further determine whether the effect SENP3-dependent deSUMOylation is associated with the increased intracellular calcium levels and calcineurin activity under hypoxia, PANC-1 cells with expression of Flag-WT NFATc3 or the NFATc3 K384R were treated with Cyclosporin A (CsA), which inhibits Ca^2+^/calcineurin signaling and thus blocks nuclear translocation of NFATc3. As a result, CsA blocked hypoxia induced Flag-WT NFATc3 or NFATc3 K384R nuclear occupancy, suggesting that deSUMOylation is not sufficient for the nuclear translocation of NFATc3 under hypoxia. Of note, CsA reversed the enhanced effect of NFATc3 K384 deSUMOylation on NFATc3 nuclear occupancy, suggesting hypoxia-induced NFATc3 dephosphorylation by calcineurin is necessary for carrying deSUMOylated NFATc3 to the nuclei (Fig. S3b). These data strongly indicated that SENP3-dependent deSUMOylation was required for NFATc3-nuclear accumulation under hypoxia.

To counteract NFATc3 nuclear localization, several kinases, such as GSK-3 and CK1, could phosphorylate NFATc3 proteins to reduce their nuclear occupancy by increasing their nuclear export [4, 25]. Furthermore, SUMOylation and deSUMOylation modulate the interactions between proteins [26–28]. We next examined whether SENP3 affected the interaction between NFATc3 and GSK-3 or CK1. Immunoprecipitation analysis showed that depletion of SENP3 augmented the interaction between WT NFATc3 and GSK-3β, but neither NFATc3 K384R mutant and GSK-3β interaction nor NFATc3 and CK1ε interaction was affected (Fig. 5d). These results indicated that SENP3 prevented the interaction of NFATc3 and GSK-3β via deSUMOylation NFATc3 K384. We next examined whether the SENP3 playing an inhibitory role in NFATc3 nuclear export could also affect NFATc3 transcriptional activity. As expected, we performed ChIP assays with an anti-NFATc3 antibody. Our data showed that SENP3 depletion significantly reduced NFATc3 occupancy at promoter region of *MYC* (Fig. 5e). In addition, hypoxia-induced transactivation of the NFATc3 responsive promoter was notably inhibited by SENP3 depletion, while the NFATc3 K384R-mediated transactivation was not evidently affected by SENP3 depletion (Fig. 5f). Taken together, these results indicated that SENP3 deSUMOylated NFATc3 at Lys384, which decreased the interaction between NFATc3 and GSK-3β and the subsequent nuclear export of NFATc3.

### DeSUMOylation of NFATc3 by SENP3 promotes PDAC cell proliferation, migration, and invasion

**Fig. 6:**
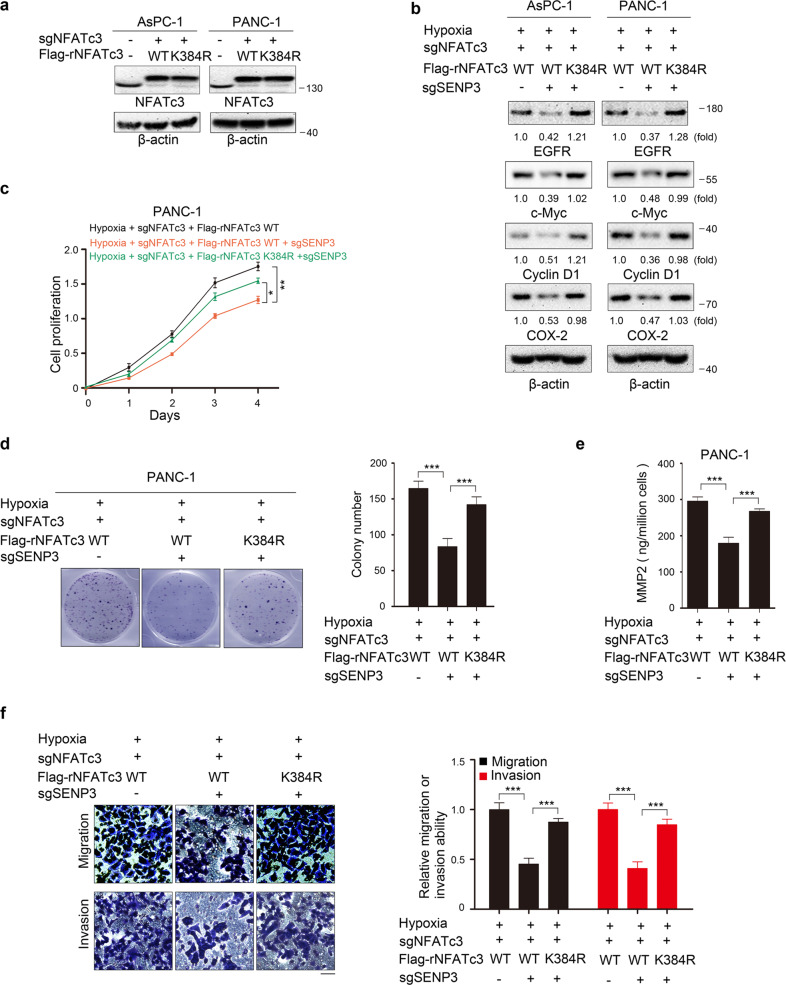
DeSUMOylation of NFATc3 by SENP3 promotes PDAC cell proliferation and metastasis in vitro. **a**, **b** Immunoblotting analyses with the indicated antibodies were performed. **a** AsPC-1 cells and PANC-1 cells with a vector expressing control sgCon or sgNFATc3 and with reconstituted expression of WT rNFATc3 or rNFATc3 K384R. **b** AsPC-1 cells and PANC-1 cells with depleted NFATc3 and reconstituted expression of WT rNFAc3 or rNFATc3 K384R were cultured with or without hypoxia for 24 h. **c**–**f** PANC-1 cells with depleted NFATc3 and reconstituted expression of WT rNFATc3 or rNFATc3 K384R were cultured with or without hypoxia. **c,** **d** Indicated PANC-1 cells were plated for the indicated periods under hypoxia before measuring cell proliferation (**c**) or for 2 weeks before counting colony numbers (**d**). Data are presented as the means ± SD from three independent experiments. *p < 0.05; **p < 0.01; ***p < 0.001. **e** Indicated PANC-1 cells were cultured in serum-free medium for 24 h under normoxia or hypoxia, and concentrations of the MMP2 in culture supernatants were measured by ELISA. Data are presented as the means ± SD from three independent experiments. ***p < 0.001. **f** The migration and invasion of the indicated PANC-1 cells were examined by the transwell assay. The membrane was photographed using a digital camera mounted onto a microscope. Scale bars, 50 μm. Data are presented as mean ± S.D. ***P < 0.001.

NFATc3 can activate downstream targets to enhance tumorigenesis. To further confirm the role of NFATc3-K384 SUMOylation in tumor progression, AsPC-1 cells and PANC-1 cells with NFATc3 depletion were reconstituted with sgRNA-resistant wild-type (WT) NFATc3 (WT rNFATc3) or the deSUMOylation mimetic mutant NFATc3 K384R (rNFATc3 K384R) (Fig. 6a). These cells with or without the expression of SENP3 sgRNA were then cultured and analyzed for NFATc3 target gene expression, proliferation, cytokines release, migration, and invasion. As a result, the expression of rNFATc3 K384R, but not WT rNFATc3, significantly reversed the inhibitory effect of SENP3 depletion on NFATc3 dependent gene expression under hypoxia (Fig. 6b). Consistently, hypoxia-induced cell proliferation (Fig. 6c, d and Fig. S4a, b), MMP2 release (Fig. 6e and Fig. S4c), and cell migration and invasion (Fig. 6f and Fig. S4d) were attenuated by SENP3 depletion in WT rNFATc3 expressing cells, but not in rNFATc3 K384R expressing cells.

**Fig. 7:**
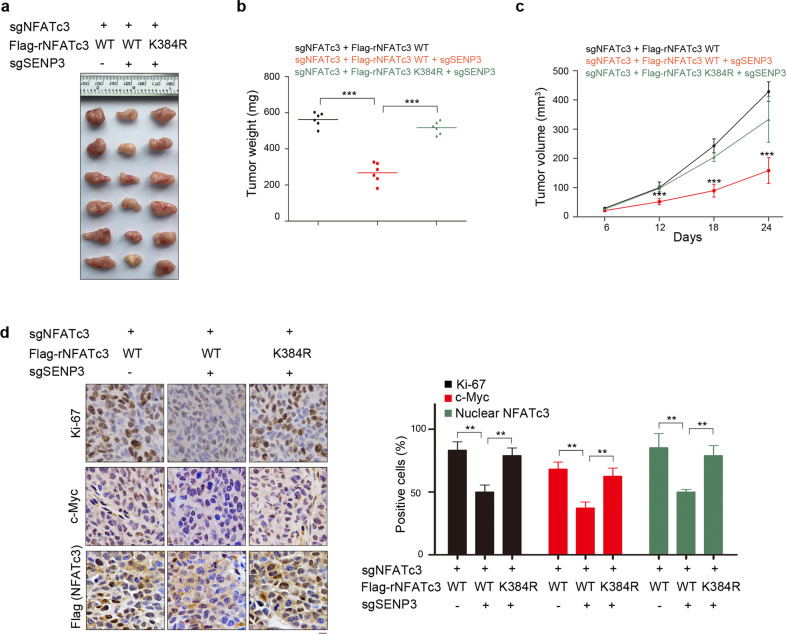
DeSUMOylation of NFATc3 by SENP3 promotes tumor growth in vivo. A total of 3 × 10^6^ control or SENP3 depleted PANC-1 cells with NFATc3 depletion and reconstituted expression of the WT NFATc3 or rNFATc3 K384R were subcutaneously injected into the athymic nude mice. Representative tumor xenografts were shown (*n* = 6 mice per group) (**a**). Tumor weights were calculated (**b**). Tumor growth was measured every other day beginning on day 6 and tumor volumes were calculated (**c**). Immunochemistry staining of the tumor sections were performed with antibodies against Ki67, c-Myc, and Flag. Representative images are shown (**d**). Scale bars, 10 μm. Data represent the means ± s.e.m. ***P < 0.001.

Moreover, the control or SENP3 depleted PANC-1 cells with NFATc3 depletion and reconstituted expression of the WT NFATc3 or rNFATc3 K384R were subcutaneously injected into the athymic nude mice. PANC-1 cells with intact SENP3 exhibited rapid tumor growth (Fig. 7a–c); in contrast, SENP3 depletion markedly inhibited tumor growth. In the meantime, the inhibitory effect of SENP3 depletion was almost compromised by rNFATc3 K384R, which corresponded with high levels of Ki67, c-Myc staining and nuclear localization of NFATc3, but not by WT rNFATc3(Fig. 7d). These results indicated that SENP3 and its inhibitory role in hypoxia-induced NFATc3 deSUMOylation could be related to pancreatic carcinoma progression.

## Discussion

The NFAT isoforms are constitutively activated in several cancer types, transactivating downstream target genes that play crucial roles in cancer development and progression [5, 8, 29]. The regulation of NFATs transcriptional activity depends mainly on post-translational modifications. Dephosphorylation is a crucial post-translational modification that exposes nuclear localization signals on NFATs, triggering their nuclear translocation and maximal transactivation [2, 4]. However, many other post-translational modifications, including acetylation, are also required for NFATs activation under various contexts [13, 30]. Here, we have uncovered that hypoxia stimulation induces the activation of NFATc3 in PDAC. Activation of NFATc3 was required for the growth, migration, and invasion of PDAC cells. More importantly, our clinical analysis indicated that high nuclear occupancy of NFATc3 was significantly associated with worse survival of PDAC patients. These results suggest that targeting NFATc3 activation may be a potential therapeutic option for treating PDAC.

PDAC is a prevalent and aggressive malignancy, and therapeutic interventions have only been marginally beneficial. This unfortunate situation is, to some degree, due to the aggressive behavior of cancer cells in the hypoxic tumor microenvironment, which highlights the urgency for designing novel therapeutic strategies to combat this deadly disease. Hypoxia will provoke multiple responses from tumor cells, including altered gene expression mediated by HIFs and drastic changes in signaling pathway. In glioblastoma and colon cancer cells, hypoxia induces the expression of the calcium channel protein, thus activating Ca^2+^ entry and NFATc3 to enhance tumor cell proliferation and invasiveness [12, 31]. In addition, it has been recently reported that the stability of SUMO2/3 protease SENP3 is enhanced through altered redox state under hypoxia and that SENP3 plays a critical role in the control of PDAC cell growth [32]. However, whether SENP3 affects tumor development through directly regulating protein deSUMOylation is unclear. In this study, we focus on the identification of additional mechanisms behind the nuclear anchorage and transcriptional activation of NFATc3 in PDAC cells under hypoxia stimulation, which would allow the development of new strategies to treat PDAC.

**Fig. 8:**
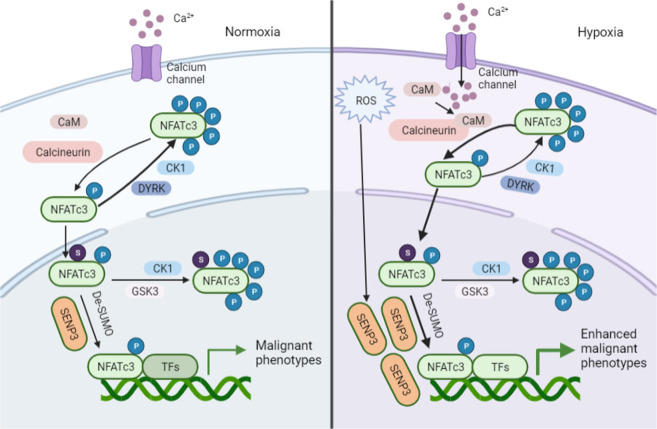
The diagram displaying the regulation of NFATc3 signaling by deSUMOylation under hypoxia. Hypoxia resulted in SENP3-mediated deSUMOylation of NFATc3 at K384, suppressing the interaction between NFATc3 and its phosphokinase GSK-3β and subsequent increasing NFATc3 nuclear occupancy. In the nuclei of PDAC cells, NFATc3 formed complexes with other factors to coordinate target gene expression, which promoted PDAC progression.

Modification by SUMOylation has been reported to play critical roles in both cytoplasmic and nuclear processes, such as transcription, nuclear transport, which ultimately contribute to regulation of cell growth, and cell cycle [15]. Moreover, a previous study has demonstrated that levels of SUMOylation of transcription factors are negatively correlated to their transcriptional activity [26]. Also, it is reported that NFATc1 isoforms c1/C can be highly SUMOylated in thymoma cells. Upon SUMOylation, NFATc1/C translocates to promyelocytic leukemia nuclear bodies and then interacts with histone deacetylases, leading to the deacetylation of histones, which ultimately suppresses chromatin activation [13]. Meanwhile, SUMOylation at Lys897 of NFATc2 is found to induce nuclear anchorage and transcriptional activation in Jurkat cells [33]. These results suggest that the exact effects of NFATs SUMOylation are context-dependent. Here, for the first time, we have demonstrated that NFATc3 can be SUMOylated. Furthermore, we have found that hypoxia stimulation could significantly promote SENP3-mediated deSUMOylation of NFATc3 at K384, suppressing the interaction between NFATc3 and its phosphokinase GSK-3β and subsequently increasing NFATc3 nuclear occupancy. In the nuclei of PDAC cells, NFATc3 could form complexes with other factors to coordinate the expression of target genes to support tumor growth (Fig. 8). Considering that the Lys384 of NFATc3 is not conserved among other members of the NFAT family, we assume that SUMOylation of Lys384 may be an underlying mechanism for isotype-specific regulation of NFATc3 nuclear occupancy and transcriptional activation under hypoxia stimulation. However, the specific mechanisms by which SUMOylation affects protein interactions between NFATc3 and SENP3 remains to be further explored.

CsA and tacrolimus, two classical inhibitors of the calcineurin–NFAT signaling axis, have shown significant anticancer activity. These immunosuppressants were found to inhibit the dephosphorylation of numerous substrates, including NFAT proteins, which may explain their neuro- and nephro-toxicity, as well as following cardiovascular and diabetic complications [34]. Therefore, tumor-specific inhibition of NFATs nuclear translocation seems to be a more promising therapeutic approach. We must remember that the physiological effects of NFAT isoforms can be cell-specific and context-dependent. Given this, further investigation in the mechanisms underlying the regulation of NFAT activation is required, so as to better understand how NFAT signaling is kept well-tuned in tumor cells to cope with environmental cues.

Here, the identified regulatory role of SENP3 in nuclear translocation of NFATc3 suggests a mechanism of how SUMOylation exerts physiological effect upon hypoxia stimuli. Our study potentially provides a molecular basis for therapeutic strategy against PDAC tumors with aberrant SENP3 expression and NFATc3 activity.

## Materials and methods

### Cell culture, transfection

PANC-1 and HEK293T cells were maintained in Dulbecco’s modified Eagle’s medium (DMEM) supplemented with 10% fetal bovine serum (FBS). AsPC-1 cells were maintained in RPMI 1640 medium supplemented with 10% FBS. All the cell lines were obtained from American Type Culture Collection (ATCC) and routinely tested for mycoplasma contamination.

Cells were plated in a six-well plate 18 h before transfection. Transfection was performed using Lipofectamine 3000 transfection reagent (Invitrogen) according to the manufacturer’s instructions. Cells lines with NFATc3 knockdown or in which the NFATc3 carried the mutation K384R or SENP3 knockdown were produced as previously described [35].

### Materials

Antibody against Flag (F1804) was obtained from Sigma. Antibody against NFATc3 (A6666) was obtained from Abclonal. Antibodies against α-Tubulin (ab210797), Hif1α (ab51608), β-actin (ab8224), stat3 (ab68153), c-jun (ab40766), cyclin D1 (ab134175), V5 (ab206566), SUMO2/3 (ab81371), SUMO3 (ab203570), CK1ε (ab270997) and Ki67 (ab92742) were obtained from Abcam. Antibodies against Lamin B1 (sc-365962) was purchased from Santa Cruz Biotechnology. Antibodies against c-Myc (#18583), SUMO2/3 (#4971 CST) and SENP3 (#5591) were obtained from Cell Signaling Technology. Antibodies against EGFR (D160292), cox2 (D223097), SENP1 (D162184), SENP2 (D19881), SENP5 (D223122), SENP6 (D162186), SENP7 (D123124) and GSK-3β (D190661) were obtained from Sangon Biotech.

CCK-8 kit was purchased from DOJINDO. Anti-FLAG® M2 Beads, EDTA-free Protease Inhibitor Cocktail, and PhosSTOP were purchased from Sigma. CsA was purchased from Selleck.

### Hypoxia exposure

Hypoxia experiments were performed in a sealed hypoxia chamber (Billups-Rothenburg, Del Mar, CA) filled with 1% O_2_, 5% CO_2_, and balanced N_2_ at 37 °C for the indicated periods of time.

### DNA constructs and mutagenesis

Polymerase chain reaction (PCR)–amplified human NFATc3 was cloned into pLenti-Flag vector. rNFATc3 WT, NFATc3 K384R, NFATc3 K434R, NFATc3 K703R, NFATc3 K1013R mutants, and SENP3 C532A were generated using a QuikChange site-directed mutagenesis kit. The primers used were shown in Table S1. sgRNA-targeting NFATc3 (5′-TCCCAGTGATTCGATGCACC-3′) and SENP3 (5′-TGTACTCTGCCCAACGGTTT-3′) were constructed into the LentiCRISPRv2 vector.

### Small interfering RNA (siRNA)

siRNA was transfected using the Lipofectamine 3000 according to the manufacturer’s instructions. The detailed information of siRNA sequences was shown in Table S2.

### Immunoprecipitation and Immunoblot Analyses

The extraction of proteins using a modified buffer (50 mM Tris-HCl (pH 7.5), 1% Triton X-100, 150 mM NaCl, 1 mM DTT, 0.5 mM EDTA, protease inhibitor cocktail, and phosphatase inhibitor cocktail) from cultured cells were followed by immunoprecipitation and immunoblot analyses using corresponding antibodies. Proteins were separated by SDS-PAGE, transferred onto PVDF membrane (Millipore), and probed with indicated antibodies.

### Nuclear-cytoplasmic fractionation

Nuclear-cytoplasmic fractionation was conducted by the Nucleo-protein Extraction Kit (Sangon, China) according to the manufacturer’s protocols.

### Immunohistochemical (IHC) staining and scoring

Human PDAC specimens and the detailed clinic-pathological information were collected from Beijing Luhe Hospital. Patient consent was obtained from all patients and the study was approved by the Ethics Committee of the Beijing Luhe Hospital. The detailed clinicopathological information were classified on the basis of the tumor classification of the American Joint Committee on Cancer staging system. The detailed information of patient characteristics was shown in Table S3. IHC assay was performed as previously reported [36]. Sections were incubated with indicated antibodies. The IHC scores was semi-quantitatively according to the staining intensity (0, no staining; 1, weak staining; 2, moderate staining; and 3, strong staining) and percentage of positive cells (1, 0%–25%; 2, 26%–50%; 3, 51%–75%; and 4, >75%). These numbers were then multiplied resulting in a score of 0–12, as described previously [37]. The IHC scores were assessed by two independent authors blinded to the clinicopathological data for all the cases.

### Mice

All the related protocols were approved by Institutional Animal Care and Use Committee of Capital Medical University. No blinding was done. The mice were randomly put into separate/groups cages for experiments. All mice were raised and fed under SPF conditions. Six-week-old male nude mice (*n* = 6 for each group, the group sizes of the animals chosen are based on the numbers we used for preliminary studies [36], which is most optimal to generate statistically significant results.) were injected with 3 × 10^6^ gene-modified PANC-1 cells in a volume of 100 μl of PBS. Injections were made subcutaneously in the left axilla at day 0. Tumor volume was calculated by the formula: *V* = *ab*^2^/2 (*a* represents the base diameter of tumor and *b* represents the corresponding perpendicular plate).

### Luciferase reporter assay

The human *MYC* promoter was inserted upstream of the luciferase reporter in pGL-3 Basic to create NFATc3-Luc. The NFATc3-Luc was used to detect NFATc3 activation. The assay was performed as previously described [38]. Briefly, cells at a density of 50–70% confluence in 24-well plates were co-transfected with NFATc3-Luc and pRL-SV40 (a plasmid encoding Renilla luciferase) using Lipofectamine 3000 (Invitrogen, USA). After transfection, the cells were cultured under hypoxic or normoxia for 12 h. Luciferase activity was assessed by the Dual-Luciferase Reporter Assay kit (Promega, USA) using a luminometer (Thermo Scientific, USA).

### Immunofluorescence

Cells grown on chambered coverslips were cultured under Hypoxic or normoxia for 12 h. Cells were washed, fixed with formaldehyde (4%), blocked, and incubated with the primary antibodies, followed by incubation with a fluorochrome-conjugated secondary antibodies. 4′,6-Diamidine-2′-phenylindole dihydrochloride (DAPI) was used to stain the nuclei. Microscope was used to acquire images(Leica, Germany).

### Cell proliferation assay

A total of 5000 cells in medium supplemented with 10% FBS were plated in 96-well plates. Ten microliter of CCK-8 solution was added into media every 24 h under hypoxia after seeding and incubated for 30 min before measurement at a wavelength of 450 nm by a microplate reader (Bio-Rad).

### Colony formation

Cells were placed into 6-well plates at a density of 500 cells/well and incubated for 2 weeks under hypoxia. After staining with 2% crystal violet for 30 min, the colonies were photographed and counted.

### Transwell assay

For transwell assay, 1 × 10^5^ cells of each group in 200 μl serum-free medium were seeded in the upper chamber without (migration) or with (invasion) Matrigel. Six hundred microliter complete medium with 10% FBS was added to the lower chamber. After 24 h (migration assay) or 30 h (invasion assay) of incubation, the non-migrating or non-invading cells were then wiped from the inside of the transwell inserts, and the penetrated cells were detected. Fix the cells with 4% formaldehyde, and stain with 0.1% crystal violet. Five randomly selected fields per filter were photographed and the total penetrated cells of each field were counted.

### Chromatin immunoprecipitation (ChIP) assay

A ChIP kit was purchased from Thermo Fisher, and ChIP assays were performed according to the manufacturer’s instruction using an anti-NFATc3 antibody. The primer pair used for PCR to amplify *MYC* promoter region was shown as following: 5′-GATCAGACACCGTCAGGGAT-3′(F), 5′-GTATGCAATAAGGTTGAAGTAAA-3' (R).

### ELISA

The levels of MMP2 in culture supernatants were measured by ELISA, following the manufacturer’s instructions (Abcam).

### Reverse transcription and Quantitative RT-PCR

Total RNA was extracted using TRIzol reagent (TaKaRa). A total of 2 μg total RNA samples were reverse transcribed to cDNA by PrimeScript RT reagent Kit (Takara). The cDNAs were quantified by Quantitative RT-PCR with SYBR Green dye (TakaRa). Data were normalized according to expression of the housekeeping gene (GAPDH) in each experiment. The primers used were shown in Table S4.

### Statistics and reproducibility

Before statistical analysis, the homogeneity of variance between groups was tested. Statistical testing was performed using the two-tailed unpaired Student’s *t*-test and *χ*^2^-test. The survival analysis was plotted by the Kaplan–Meier method and were compared by the log-rank test. All experiments were performed at least three times unless otherwise indicated. N numbers are indicated in the figure legends. Data represent the mean ± SD. Statistical significance was defined as *p* < 0.05.

## Supplementary information

Supplemental material

Supplemental table 1

Supplemental table 2

Supplemental table 3

Supplemental table 4

Reproducibility checklist

Supplemental figure 1

Supplemental figure 2

Supplemental figure 3

Supplemental figure 4

Soure data

## Data Availability

Raw data from all figures and supplementary figures are included in the source data file. Additional data related to this paper may be requested from the corresponding author. Source data are provided with this paper.
