# Supplementary material for: Hypoxia-induced NFATc3 deSUMOylation enhances pancreatic carcinoma progression
Source: Cell Death Dis. 2022 Apr 28;13(4):413. doi: 10.1038/s41419-022-04779-9 (PMC9050899; doi:10.1038/s41419-022-04779-9)
Supplement: Supplementary file 1 — Supplemental material [file 41419_2022_4779_MOESM1_ESM.docx]

**Supplemental Figure Legends**

**Fig. S1 Nuclear localized NFATc3 is positively correlated with Hif1α and poorer prognosis in PDAC specimens**

a. Expression of HIF1α in 60 samples of human PDAC tissues and matched adjacent normal tissues was measured by IHC staining with an anti-HIF1α antibody. Representative images are shown. Scale bars, 10 μm.

b. Comparative analysis of HIF1α expression between PDAC tissues and matched adjacent normal tissues (***P < 0.001).

c. Kaplan–Meier plots and p-values of the log-rank test for comparing survivals of PDAC patients with high (staining score, 5–12) and low (staining score, 0–4) expression of HIF1α.

d. Semi-quantitative scoring between HIF1α and nuclear NFATc3 were performed (Pearson’s correlation test; R = 0.6966, P < 0.001).

e. PANC-1 and AsPC-1 cells were stimulated with or without hypoxia for the indicated time. The mRNA levels of *EGRF*, *MYC*, *CCND1* and *COX2* in the indicated cells were measured by qRT-PCR. The results were statistically calculated from three independent experiments. Data are presented as mean ± S.D. ***P < 0.001

**Fig. S2 NFATc3 is deSUMOylated by SENP3 under hypoxia**

a. Expression of SENP3 in 60 samples of human PDAC tissues and matched adjacent normal tissues was measured by IHC staining with an anti-SENP3 antibody. Representative images are shown. Scale bars, 10 μm.

b. Comparative analysis of SENP3 expression between PDAC tissues and matched adjacent normal tissues (***P < 0.001).

c. Semi-quantitative scoring between HIF1α and SENP3 were performed (Pearson’s correlation test; R = 0.8288, P < 0.001).

d. Kaplan–Meier plots and p-values of the log-rank test for comparing survivals of PDAC patients with high (staining score, 5–12) and low (staining score, 0–4) expression of SENP3.

**Fig. S3 NFATc3 K384 deSUMOylation by SENP3 increases nuclear translocation of NFATc3 by decreasing its interaction with GSK-3β**

a. Semi-quantitative scoring between SENP3 and nuclear NFATc3 were performed (Pearson’s correlation test; R = 0.7377, P < 0.001).

b. PANC-1 cells that stably expressing Flag-WT NFATc3 or Flag-NATc3 K384R were pretreated with CsA (1.0 µM) for 1 h before being cultured for 24 h under hypoxia.

**Fig. S4 DeSUMOylation of NFATc3 by SENP3 promotes PDAC cell proliferation and metastasis in vitro**

(a - d) AsPC-1 cells with depleted NFATc3 and reconstituted expression of WT rNFATc3 or rNFATc3 K384R were cultured with or without hypoxia.

(a and b) Indicated AsPC-1 cells were plated for the indicated periods under hypoxia before measuring cell proliferation (a) or for two weeks before counting colony numbers (b). Data are presented as the means ± SD from three independent experiments. *p < 0.05; **p < 0.01; ***p < 0.001.

c. Indicated AsPC-1 cells were cultured in serum free medium for 24 h under normoxia or hypoxia, and concentrations of the MMP2 in culture supernatants were measured by ELISA. Data are presented as the means ± SD from three independent experiments. ***p < 0.001.

d. The migration and invasion of the indicated AsPC-1 cells was examined by the transwell invasion assay. The membrane was photographed using a digital camera mounted onto a microscope. Scale bars, 50 μm. Data are presented as mean ± S.D. ***P < 0.001.
