# Supplementary material for: Hypoxia-induced NFATc3 deSUMOylation enhances pancreatic carcinoma progression
Source: Cell Death Dis. 2022 Apr 28;13(4):413. doi: 10.1038/s41419-022-04779-9 (PMC9050899; doi:10.1038/s41419-022-04779-9)
Supplement: Supplementary file 2 — Supplemental table 1 [file 41419_2022_4779_MOESM2_ESM.docx]

Table S1. Sequences of mutated primers

| **Primer** | **Sequence** | **Base number** |
| --- | --- | --- |
| WT rNFATc3-F | CTCATGCATTTTACCAGGTACACCGG ATAACGGGGAAGACAGTCG | 45 |
| WT rNFATc3-R | CGACTGTCTTCCCCGTTATCCGGTGT  ACCTGGTAAAATGCATGAG | 45 |
| NFATc3 K384R-F | TGATCACCACATGAATCTTTCCTTAA  AGGATACTGAGATCCAAG | 44 |
| NFATc3 K384R-R | CTTGGATCTCAGTATCCTTTAAGGAAA  GATTCATGTGGTGATCA | 44 |
| NFATc3 K434R-F | GAGTTTTAGGTTGCACTTCTATTCTCA  GTTCACATTGTCCAAAATGA | 47 |
| NFATc3 K434R-R | TCATTTTGGACAATGTGAACTGAGAAT  AGAAGTGCAACCTAAAACTC | 47 |
| NFATc3 K703R-F | TCTCTGTGTTCTTGCCTCATCAAAACT  GGTGTATAAGTAAAACGT | 45 |
| NFATc3 K703R-R | ACGTTTTACTTATACACCAGTTTTGATG  AGGCAAGAACACAGAGA | 45 |
| NFATc3 K1013R-F | TCTGGTTCAGGTCTAATGCTCACAGTT  GCCCCATCA | 36 |
| NFATc3 K1013R-R  SENP3 C532A-F  SENP3 C532A-R | TGATGGGGCAACTGTGAGCATTAGACC  TGAACCAGA  CTGCAACACAAAAGCACCAGCGTCAC  TGTCATTATTCTGC  GCAGAATAATGACAGTGACGCTGGTG  CTTTTGTGTTGCAG | 36  40  40 |
