# Supplementary material for: Hypoxia-induced NFATc3 deSUMOylation enhances pancreatic carcinoma progression
Source: Cell Death Dis. 2022 Apr 28;13(4):413. doi: 10.1038/s41419-022-04779-9 (PMC9050899; doi:10.1038/s41419-022-04779-9)
Supplement: Supplementary file 3 — Supplemental table 2 [file 41419_2022_4779_MOESM3_ESM.docx]

Table S2. Sequence of siRNA sequences

| **Gene** | **Sequence** | |  |  |  |
| --- | --- | --- | --- | --- | --- |
| siSENP1-F |  | AUUCAUGUAGAAAUUGAUGAU | | | |
| siSENP1-R |  | CAUCAAUUUCUACAUGAAUAU | | | |
| siSENP2-F |  | UUAAUGUCUGAAUAUCUCCUC | | | |
| siSENP2-R |  | GGAGAUAUUCAGACAUUAAAG | | | |
| siSENP3-F |  | ACGUUUGAAGGAAUUCGUCCA | | | |
| siSENP3-R |  | GACGAAUUCCUUCAAACGUAU | | | |
| siSENP5-F |  | AAGAAAGAGCUUCCUUUAGUG | | | |
| siSENP5-R |  | CUAAAGGAAGCUCUUUCUUGG | | | |
| siSENP6-F |  | ACAAACUUGUUUUCCUUUCUU | | | |
| siSENP6-R |  | GAAAGGAAAACAAGUUUGUCA | | | |
| siSENP7-F |  | AUCAACUUCUGAACAAGUCCA | | | |
| siSENP7-R |  | GACUUGUUCAGAAGUUGAUUG | | | |
