# Supplementary material for: Hypoxia-induced NFATc3 deSUMOylation enhances pancreatic carcinoma progression
Source: Cell Death Dis. 2022 Apr 28;13(4):413. doi: 10.1038/s41419-022-04779-9 (PMC9050899; doi:10.1038/s41419-022-04779-9)
Supplement: Supplementary file 4 — Supplemental table 3 [file 41419_2022_4779_MOESM4_ESM.docx]

Table S3. Clinicopathological factors in 60 PDAC patients

| **Characteristics** | | **n =60** |
| --- | --- | --- |
| **Age** |  |  |
| ≤ 60 |  | 23 |
| ＞ 60 |  | 37 |
| **Sex** |  |  |
| Male |  | 32 |
| Female |  | 28 |
| **Greatest tumor diameter (cm)** | | |
| ＜ 4 |  | 22 |
| ≥ 4 |  | 38 |
| **Lympy node involvement** | | |
| Negative |  | 33 |
| Positive |  | 27 |
| **Histologic grade** | | |
| ≤ II |  | 35 |
| ＞ II |  | 25 |
| **Stage** |  |  |
| ≤ II |  | 47 |
| ＞ II |  | 13 |
