# Supplementary material for: Hypoxia-induced NFATc3 deSUMOylation enhances pancreatic carcinoma progression
Source: Cell Death Dis. 2022 Apr 28;13(4):413. doi: 10.1038/s41419-022-04779-9 (PMC9050899; doi:10.1038/s41419-022-04779-9)
Supplement: Supplementary file 5 — Supplemental table 4 [file 41419_2022_4779_MOESM5_ESM.docx]

Table S4. Sequence of primers

| **Primer** | **Sequence** | **Base number** |
| --- | --- | --- |
| *SENP3-*F | CATGTACTCTGCCCAACGGT | 20 |
| *SENP3-*R  *EGFR-*F  *EGFR-*R  *MYC-*F  *MYC-*R  *CCND1-*F  *CCND1-*R  *COX2-*F  *COX2-*R | CCCCGATGCTGCACACATTG  AGGCACGAGTAACAAGCTCAC  ATGAGGACATAACCAGCCACC  GTCAAGAGGCGAACACACAAC  TTGGACGGACAGGATGTATGC  GCTGCGAAGTGGAAACCATC  CCTCCTTCTGCACACATTTGAA  TAAGTGCGATTGTACCCGGAC  TTTGTAGCCATAGTCAGCATTGT | 20  21  21  21  21  20  22  21  23 |
| *GAPDH*-F | GGAGCGAGATCCCTCCAAAAT | 21 |
| *GAPDH*-R | GGCTGTTGTCATACTTCTCATGG | 23 |
