# Supplementary figures and images for: Hypoxia-induced NFATc3 deSUMOylation enhances pancreatic carcinoma progression
Source: Cell Death Dis. 2022 Apr 28;13(4):413. doi: 10.1038/s41419-022-04779-9 (PMC9050899; doi:10.1038/s41419-022-04779-9)

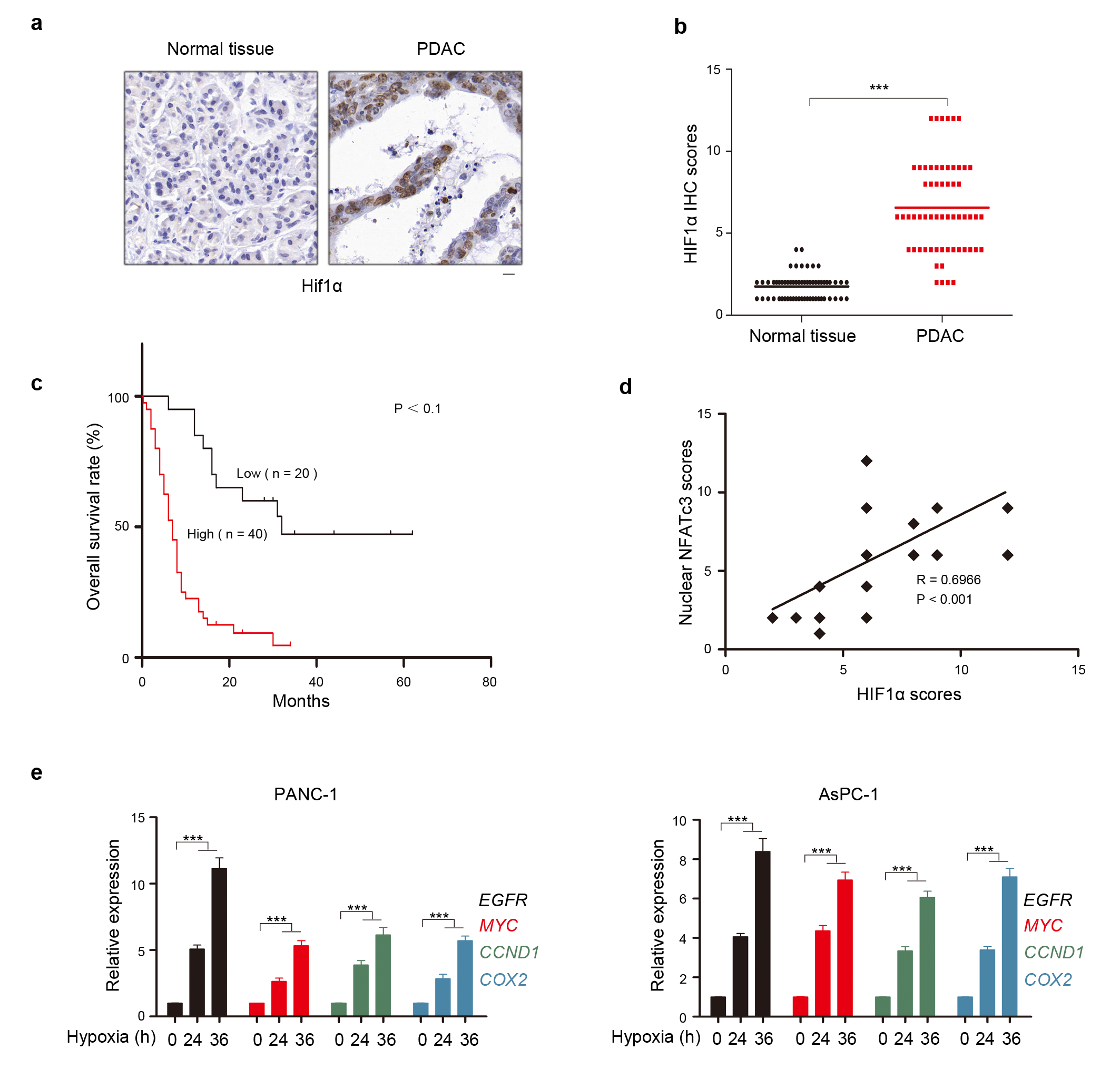

Supplement: Supplementary file 7 — Supplemental figure 1 [file 41419_2022_4779_MOESM7_ESM.tif]

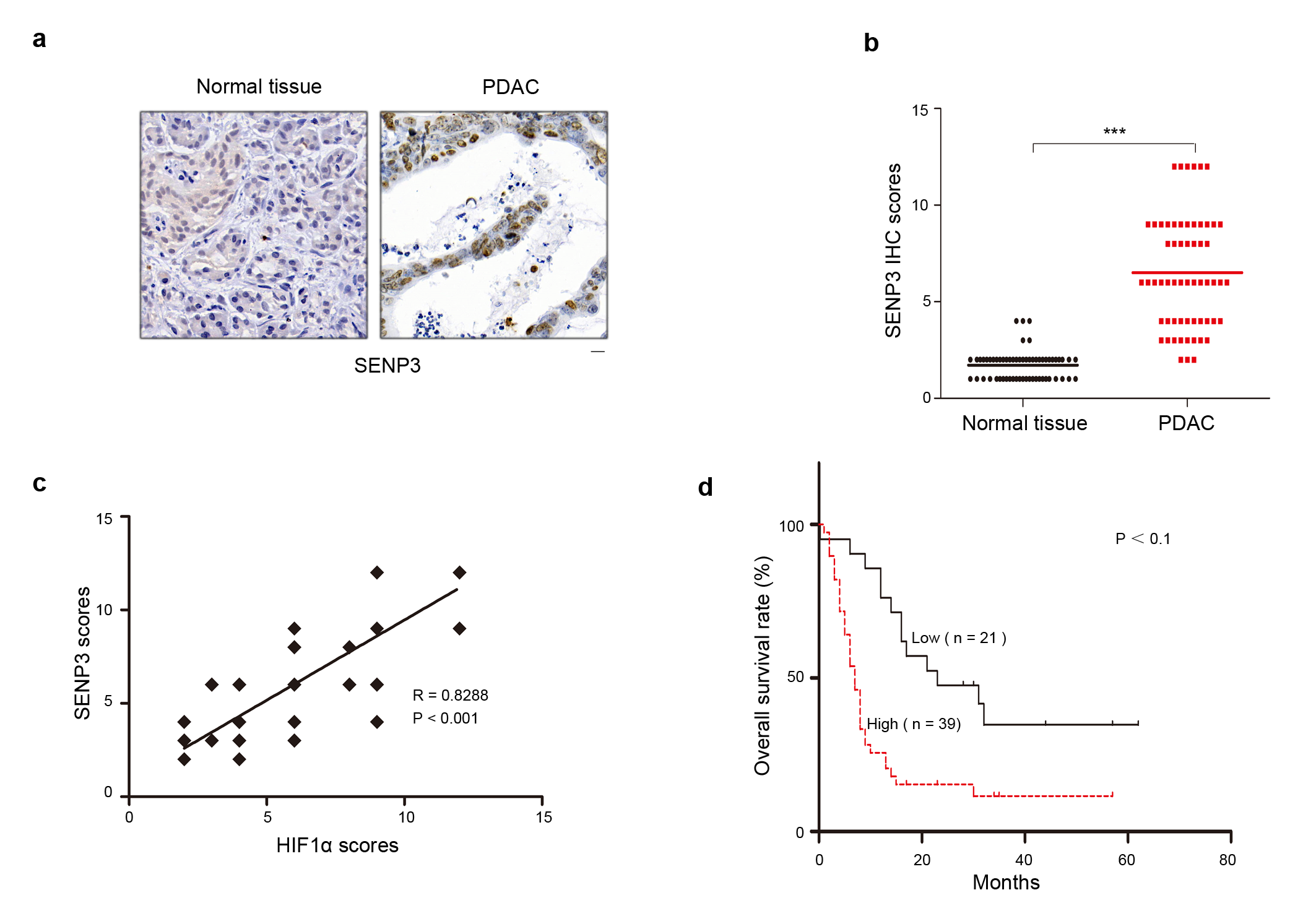

Supplement: Supplementary file 8 — Supplemental figure 2 [file 41419_2022_4779_MOESM8_ESM.tif]

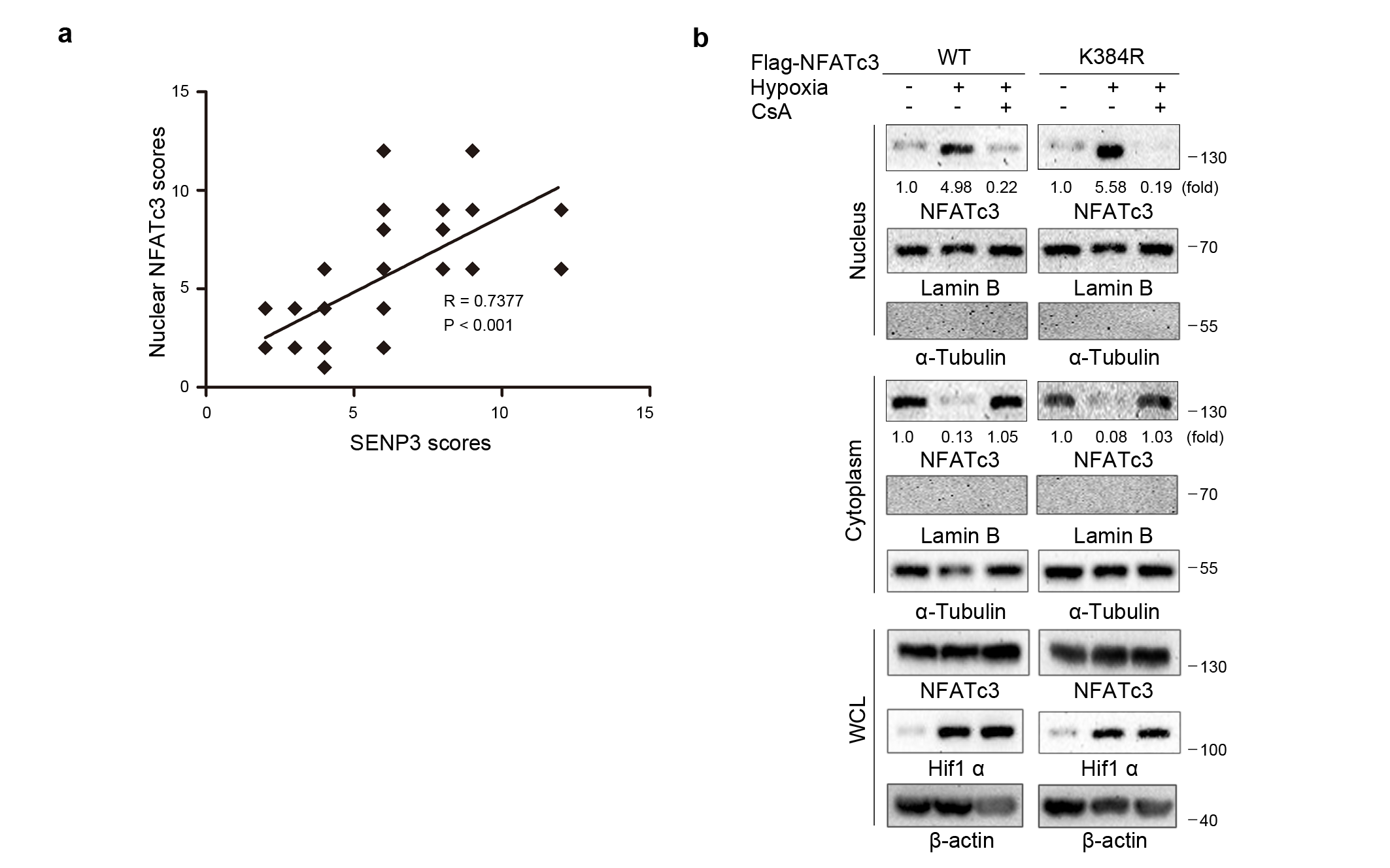

Supplement: Supplementary file 9 — Supplemental figure 3 [file 41419_2022_4779_MOESM9_ESM.tif]

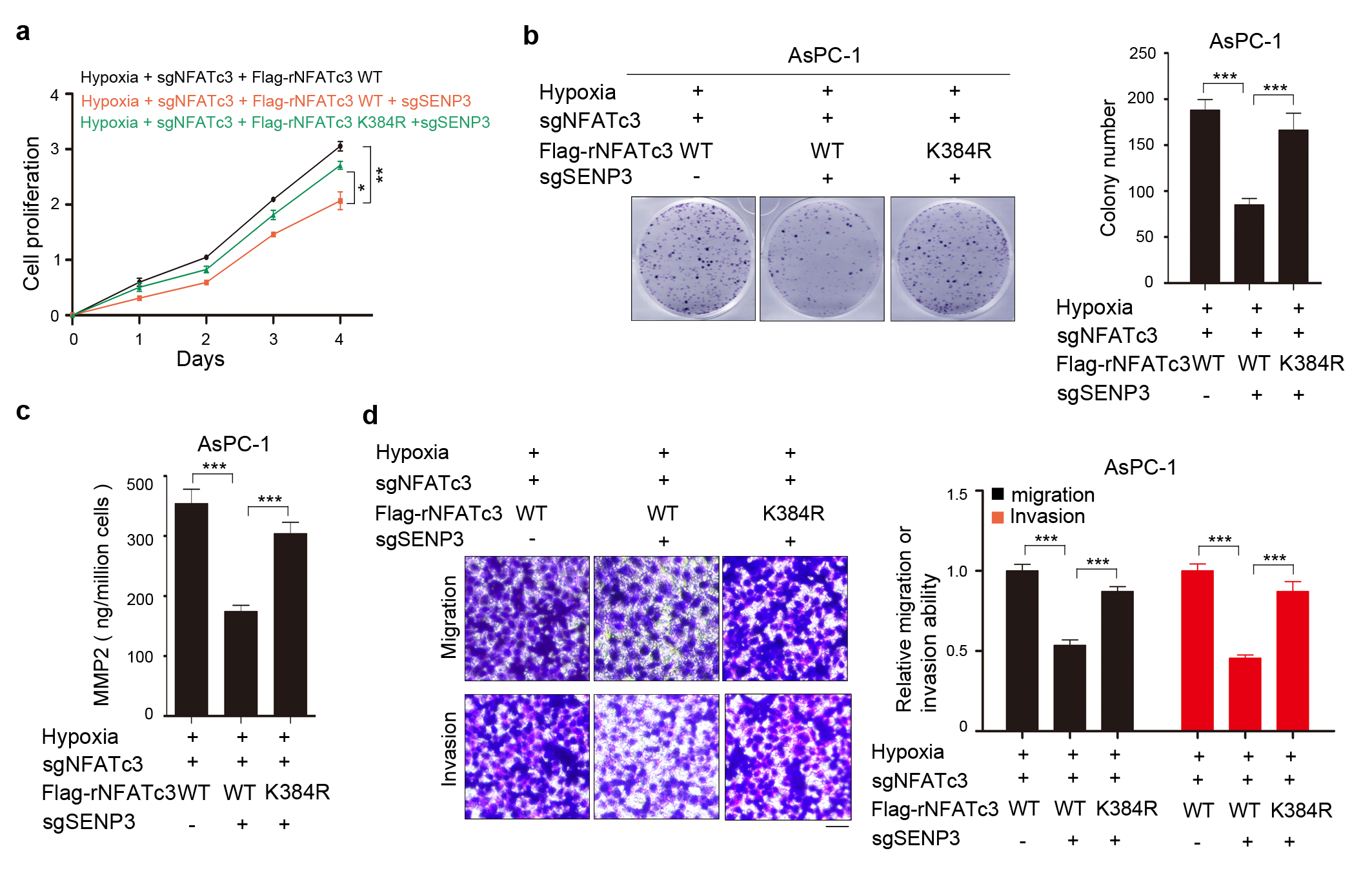

Supplement: Supplementary file 10 — Supplemental figure 4 [file 41419_2022_4779_MOESM10_ESM.tif]
